# Addressing the Problem of Brain Death Misdiagnosis: A ‘Just’ Evaluation of a Difficult Problem

**DOI:** 10.1017/jme.2025.10108

**Published:** 2025

**Authors:** Christopher A. DeCock, Cody F. Feikles

**Affiliations:** 1 Essentia Health West Region, Fargo, ND, United States; 2 University of North Dakota School of Medicine & Health Sciences, Grand Forks, ND, United States; 3 OSF St. Francis Medical Center College of Nursing, Peoria, IL, United States; 4 Georgetown University Kennedy Institute of Ethics, Washington, D.C., United States

**Keywords:** brain death, law, Uniform Determination of Death Act (UDDA), AAN 2023 DNC Guidelines, death by neurological criteria, whole brain death, dead donor rule

## Abstract

In their article, Drabiak et al. review the state laws and ethical debates related to the determination of death by neurologic criteria, analyze the recent 2023 American Academy of Neurology practice guidelines, and make policy recommendations. We call this review ‘just’ because the article correctly focuses on the chief ethical, legal, and medical issue in this debate — namely whether patients declared dead by neurological criteria are actually dead, along with the need to improve integrity, honesty, trust, and residency education and training to reduce moral distress and achieve moral certainty in declaring patients dead, initiating organ procurement, and communicating these realities to patient families/surrogates. As the authors invite the reader to comprehend, it should no longer be considered a minority or fringe opinion that determinations of brain death are rife with false positives, inadvertent misdiagnoses, violations of informed consent, and, ultimately, dissent from the law. For the sake of justice, one would do well to heed these words.

In their article, Drabiak et al. review the state laws and ethical debates related to brain death determination/death by neurologic criteria (DNC), analyze the recent 2023 American Academy of Neurology (AAN) practice guidelines, and make policy recommendations.^1^ They highlight how the law, the Uniform Determination of Death Act (UDDA), and practice guidelines are not in sync, resulting in false positive and/or inadvertently misdiagnosed cases of brain death. This is because, as the authors point out, the law requires “irreversible cessation of all functions of the entire brain,” whereas the AAN guidelines state that death can be determined *even when some brain functioning is still present.* ^2^ Moreover, whereas the law supports patients’ rights to accept or refuse medical exams and interventions under the doctrine of informed consent, they highlight how the “AAN’s recommendations conflict with the legal requirement for informed consent.” Furthermore, the authors cite compelling research revealing the sore lack of consistency, familiarity, and competency among both medical professionals and students regarding assessing for and determining death by neurological criteria, resulting in “inadvertent misdiagnosis from unintentional errors.” In the end, they recommend upholding the existing law by improving medical testing and standardizing procedures in the determination of brain death. Additional concerns related to informed consent and accommodations are discussed, but no solutions are provided other than a reminding of the extant legal grounds for supporting informed consent/dissent for brain death evaluations.

We call this review “just” because Drabiak et al. focus on the chief ethical, legal, and medical issue in this debate — namely whether the patient declared dead by neurological criteria is *actually* dead. As an Observer for the Uniform Law Commission (ULC)’s Revision for the UDDA, DeCock witnessed firsthand the various justifications for changing the legal standard of whole brain death (i.e., irreversible loss of function of all parts of the brain) to partial brain death (i.e., permanent loss of function of some parts of the brain). The proposals to eliminate brain death as a legal standard or to improve testing requirements, and thereby achieve moral certitude in pronouncing patients dead, were dismissed out of hand *because they would decrease the number of organs available for organ transplantation.* For those of us who have studied the genesis of the concept of brain death, this calls to mind part of the rationale behind the 1968 Harvard Ad Hoc committee, which stated that the need to define brain death was in part due to the need to free up beds within intensive care units and to advance the cause of organ transplantation.^3^ And as Edmund Pellegrino opined in 2008 on the dilemma of brain death, “the vexed issue of social versus individual good arises and sharp differences of opinion between and among interested parties seem unavoidable.”^4^ This was true both in 1968, during the 2008 President’s Council on Bioethics, and was certainly true in the 2023 UDDA revision attempts.

Drabiak et al. do not fall prey to these temptations. They clearly demonstrate that the current clinical guidelines have undoubtedly led to false positives and assessment inconsistencies and must be improved. This sentiment was echoed by the American College of Physicians (ACP) in 2023.^5^ Although many believe that such an opinion is merely an objection of the minority, the actual statements received by the ULC drafting committee tell a different story with 82% of organizational comments opposed to a partial brain death standard, proposed by the AAN as a “brain-as-a-whole” standard (not to be confused with the current “whole-brain” legal standard of death).^6^ Additionally, it is worth recalling that the ACP is the second largest physician organization behind the American Medical Association (AMA) and is four times larger than the AAN. The AMA has yet to comment.

Rather than focusing on what benefits might arise from declaring a patient brain dead, the authors focus on the patients themselves citing the importance of honoring intrinsic human dignity. They rightly argue that any violation of a patient’s intrinsic human dignity, even supposedly for the common good, is unacceptable. In medicine, physicians have never believed that it was acceptable to kill one patient for the good of another. Such utilitarian rationale would violate the Dead Donor Rule, which is vitally important to safeguard the vulnerable patients who are being evaluated for brain death. Critically, they also discuss the importance of integrity and honesty, trust, non-maleficence, and moral certainty surrounding declaring patients dead, initiating organ procurement, and communicating these realities to patient families/surrogates. Moreover, they cite the scourge of moral distress that the problems with the current approaches to brain death determination have wrought on practitioners and families alike. In a time when medicine has lost so much of the public trust, unless we improve transparency and certainty in our ability to correctly determine someone to be dead, we will continue to see a decrease in patients willing to be vital organ donors.

In addition to improving testing, the authors also provide some suggestions to ensure that clinician evaluation is free from misdiagnosis. Education has always been key to improving the accuracy of a diagnosis, and Drabiak et al. note this, discussing both residency education and didactic learning and simulatio — both tried and true tools available to the teaching clinician. Although many states do not specify *who* may determine someone to be brain dead, the authors agree with the AAN that credentialing could result in higher degrees of certitude that the clinician is aware of the standard practice of medicine and is thus prepared to rule out confounders and exhaust additional treatment options prior to moving to a determination of brain death. However, it is unclear whether such training would consider the importance of improved testing or rather reiterate the oft-repeated claim that the 2023 AAN Guidelines are good enough. Nonetheless, the authors deftly remind us that “institutions that do not provide adequate training and oversight for physicians that perform DNC exams or lack institutional policies on guidance for physicians to adhere to legal and accepted medical standards may also face potential corporate negligence claims.”

As the authors invite the reader to comprehend, it should no longer be considered a minority or fringe opinion that determinations of brain death are rife with false positives, inadvertent misdiagnoses, violations of informed consent, and, ultimately, dissent from the law — both in their opposition to the legally accepted definition of death according to neurological criteria (i.e., whole-brain-death) and the subsequent violations of the dead donor rule for the sake of organ procurement. Decreasing these problems in adherence with the law and ethical norms is the main thrust of the authors. As Grisez and Boyle stated in 1979, “where experts disagree, those who are not experts have reason to doubt and have no basis to proceed with confidence in a matter which requires certitude beyond a reasonable doubt, when it is not absolutely necessary to proceed. And although it may be necessary to ignore the needs of some bodies, even live ones, it is never necessary to consider any body dead who might be alive.”^7^ For the sake of justice, one would do well to heed these words.

## References

[1] M. Fitzsimmons, K. Drabiak, and P. Shetty, “Addressing the Problem of Brain Death Misdiagnosis,” Journal of Law, Medicine, & Ethics 53, no.2 (2025): 244–253. 10.1017/jme.2025.10107 PMC12355472 40432411

[2] National Conference of Commissioners on Uniform State Laws, Uniform Determination of Death Act (U.S. Government Printing Office, 1981): 5; D.M. Greer et al., “Pediatric and adult brain death/death by neurologic criteria consensus guideline: report of the AAN Guidelines Subcommittee, AAP, CNS, and SCCM,” *Neurology* 101, no. 24 (2023): 1112–1132, 10.1212/WNL.0000000000207740. PMC10791061 37821233

[3] Ad Hoc Committee of the Harvard Medical School to Examine the Definition of Brain Death, “A Definition of Irreversible Coma: Report of the Ad Hoc Committee of the Harvard Medical School to Examine the Definition of Brain Death,” Journal of the American Medical Association 205, no. 6 (1968): 337–340, 10.1001/jama.1968.03140320031009. 5694976

[4] E.D. Pellegrino , Personal Statement of Edmund D. Pellegrino, M.D. in Controversies in the Determination of Death: A White Paper by the President’s Council on Bioethics (President’s Council on Bioethics, December 2008), 119.

[5] American College of Physicians, “Statement to the Uniform Law Commission regarding updating of the Uniform Determination of Death Act (UDDA),” June 7, 2023. Accessed August 7, 2023. https://www.uniformlaws.org/viewdocument/comments-various-authors?CommunityKey=a1380d75-62bc-4a5b-ba3a-e74001a9ab57&tab=librarydocuments#MainCopy_ctl21_DocumentFileListView_DocumentTitleLink_40

[6] For example, the American College of Physicians, Association of American Physicians and Surgeons, United States College of Catholic Bishops, Christian Medical and Dental Association and the Arc (representing the disability community). These and others may be found on the Uniform Law Commission’s website: Uniform Law Commission. *Determination of Death Committee.* Documents. Last reviewed on March 6, 2024. https://www.uniformlaws.org/committees/community-home/librarydocuments.

[7] G. Grisez and J.M. Boyle, “Definition of Death” in Life and Death with Liberty and Justice: A Contribution to the Euthanasia Debate (University of Notre Dame Press, 1979): 78.

